# A Rare Case of a Plunging Ranula in a 42-Year-Old Male Patient: A Case Report

**DOI:** 10.7759/cureus.68077

**Published:** 2024-08-29

**Authors:** Yanko G Yankov, Ralitsa V Yotsova, Nikolay I Nikolaev, Lyuben L Stoev, Lachezar I Plachkov, Simeon N Dimanov, Martina G Stoeva

**Affiliations:** 1 Maxillofacial Surgery, University Hospital St. Marina, Varna, BGR; 2 General and Operative Surgery, Мedical University - Varna Prof. Dr. Paraskev Stoyanov, Varna, BGR; 3 Oral Surgery, Мedical University - Varna Prof. Dr. Paraskev Stoyanov, Varna, BGR; 4 Maxillofacial Surgery, Мedical University - Varna Prof. Dr. Paraskev Stoyanov, Varna, BGR; 5 General and Clinical Pathology, Forensic Medicine, and Deontology, Мedical University - Varna Prof. Dr. Paraskev Stoyanov, Varna, BGR; 6 Imaging Diagnostics and Interventional Radiology, Мedical University - Varna Prof. Dr. Paraskev Stoyanov, Varna, BGR; 7 Imaging Diagnostics, University Hospital St. Marina, Varna, BGR

**Keywords:** oral diseases, pseudocyst, sublingual salivary glands, neck pathology, recurrent diseases, neck surgery, swelling of the neck, maxillofacial surgery, thyroglossal duct cysts, plunging ranula

## Abstract

Plunging ranulas are rare retention pseudocysts of the major salivary glands, most often of the sublingual gland, and usually occur in individuals from the first to the sixth decade of life with female predominance. Given their similar location and physical and imaging characteristics to thyroglossal cysts, distinguishing the two lesions is often a differential diagnostic dilemma even for the experienced physician. This case report presents a 42-year-old man for whom a preliminary diagnosis of a thyroglossal duct cyst was made based on a physical examination. A neck ultrasound was performed and the lesion was surgically excised. However, pathological analysis revealed a plunging ranula of a salivary gland. A contrast-enhanced computed tomography (CT) of the neck was performed. It showed close proximity of the lesion to the right sublingual salivary gland. Because of this, it was assumed that the gland was associated with the occurrence of his condition, and the patient was offered a complete sialoadenectomy. However, the patient refused this plan of treatment. Approximately seven months later there was a recurrence for which he was operated on again. Despite the warning of a high probability of recurrence, the patient categorically refused sialadenectomy.

## Introduction

The term "ranula" refers to a retention cyst of the sublingual or the submandibular salivary gland. It occurs as a result of obstruction or trauma of the glandular drainage channels - the ducts of Rivinus and less often the duct of Bartholin in the case of the sublingual gland and extremely rarely in the Wharton’s duct in the submandibular gland [1]. Subsequently, there is extravasation and accumulation of saliva in the soft tissues of the floor of the oral cavity [1,2]. Clinically, bluish and transparent cystic formation and gradual, progressive growth and expansion into surrounding tissues are observed [1,2].

The frequency of ranulas is 0.2-0.9 per 1000, thus representing only 6% of all salivary gland cysts [3,4]. According to their clinical appearance, there are three types of ranulas: oral (superficial), plunging (cervical, deep), and mixed type, which is a combination of the previous two [5]. Plunging ranula is often seen in combination with oral-type ranula. The extravasated saliva accumulates between the cervical fascial layers and the hypoglossal muscles, herniates the mylohyoid muscle, and passes into the submandibular space, and sometimes in the direction of the parapharyngeal space [2,3,6].

The diagnosis is sometimes easily achieved on clinical examination, but distinguishing between superficial and deep types of ranula can be a diagnostic challenge. In these cases, imaging such as ultrasound, magnetic resonance imaging (MRI), and computed tomography (CT) are required to confirm the diagnosis and provide data on the extent of the process [1,3]. Differential diagnosis includes salivary gland cysts, vascular malformations, hemangiomas, lymphangiomas, and other cysts involving the floor of the oral cavity (such as dermoid cysts), submandibular space, parapharyngeal space, and neck (such as thyroglossal duct cyst and branchial cleft cyst) [3].

This case report, in order to add valuable information to the current medical knowledge about the diagnosis and treatment of patients with plunging ranula, shares our experience by presenting a patient with this relatively rare condition.

## Case presentation

We present a 42-year-old male Bulgarian citizen who, in September 2023, visited a maxillofacial surgeon at the Clinic of Maxillofacial Surgery, University Hospital St. Marina (Varna, Bulgaria) with a complaint of a painless swelling on the front of his neck that was gradually increasing in size and causing him discomfort for about three months. No voice hoarseness was reported. The patient did not report any history of trauma in the head and neck region. The patient had no proven allergies to food and medications, no previous surgical interventions, and no family medical history. He had been medically treated for hypertension with Candesartan and Bisoprolol for two years and maintained a normal blood pressure level.

Physical examination revealed the presence of a painless, soft tumor with size of about 6x3 cm in the upper third of the midline of the neck anteriorly, above the hyoid bone, which remained immobile upon swallowing. The lesion was not fixed to the surrounding tissues, was not tender at palpation, and did not cause any restriction of neck movement. The overlying skin was unchanged. No cervical lymphadenopathy was palpated. Upon intraoral examination, no pathological changes were observed.

The patient underwent an ultrasound of the neck as a routine procedure, which visualized a large septated cystic collection in the submental space, irregular in shape and measuring about 60/20 mm. The described finding showed a mild mass effect and no involvement of the surrounding soft tissues.

Thus, on the basis of the physical and ultrasound findings, a preliminary diagnosis of a median neck cyst was made, and in October of the same year, the patient was hospitalized for surgical removal of the lesion. Under general anesthesia with orotracheal intubation, after thorough antiseptic of the operative field and infiltration of 2% Lidocaine with Adrenaline 1:100000 for hemostasis through a horizontal incision of the skin and subcutaneous tissue in the upper cervical third anteriorly, above the existing swelling, a very thin cyst-like mass was reached. It had a very thin and friable lining and was not connected to the hyoid bone. The lesion was excised in visibly clear margins and sent for histological examination. Hemostasis was achieved with tissue cauterization. A silicone drain was placed, and the wound was sutured in layers with resorbable polyfilament for muscle, fascia, and subcutaneous tissue and with non-resorbable monofilament for skin. Perioperative prophylaxis with Cefazolin was administered intravenously, 2 g three times a day for three days.

**Figure 1:**
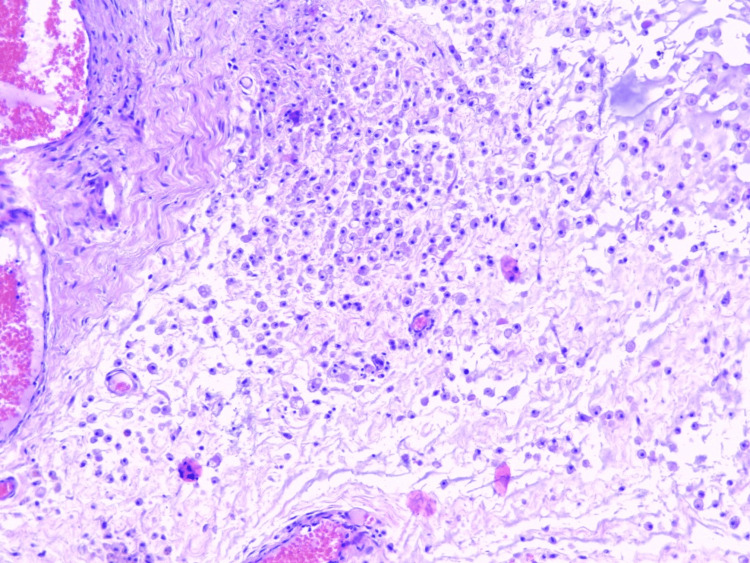
Photomicrograph of the pseudocystic lesion showing soft tissues, dissected by mucin and numerous macrophages. No epithelial lining is present in any focus of the lesion (H&E x10)

**Figure 2:**
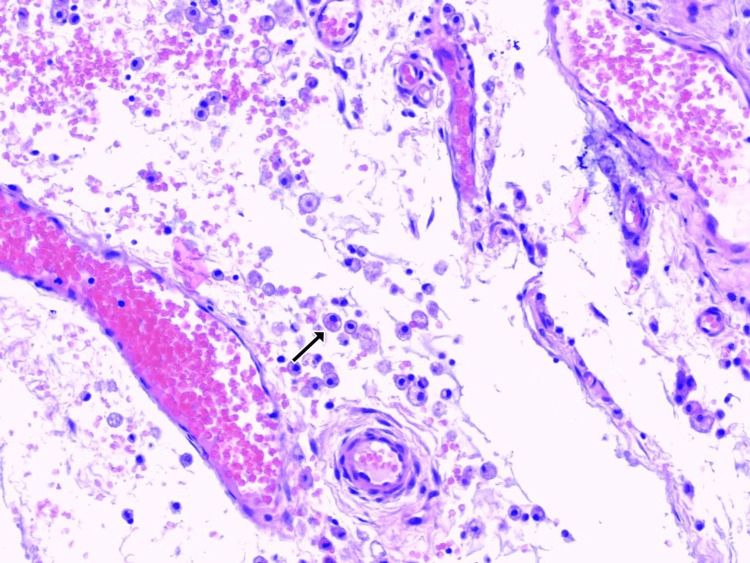
Collection of numerous muciphages (arrow) in the soft tissues and around vasculature (H&E x20)

The histological examination revealed a pseudocyst filled with mucin and numerous muciphages. Although the whole lesion was submitted for Paraffin embedding and histological examination and deep sections of the Paraffin blocks were performed, there was no evidence of epithelial lining on any of the slides (Figures 1, 2). No evidence of glandular parenchyma was present. The final pathological report described a mucinous pseudocystic lesion compatible with ranula.

On the basis of the patient's complaints, the physical examination, the data from the ultrasound of the neck, and the pathological analysis, the diagnosis of plunging ranula was made.

The postoperative period was uneventful without significant complaints from the patient. The drain was removed on day 3 postoperatively. The patient was discharged in good general condition, with an uncomplicated postoperative wound and stable vital signs. He was followed up regularly for three months. The wound showed signs of complete healing in the sixth week.

At the end of March 2024 (approximately seven months after the operation), on the occasion of a new swelling in the same area and with the same characteristics, showing progressive enlargement over several weeks, the patient visited the maxillofacial surgeon again. On physical examination, a cyst-like mass of about 5 cm in diameter was visualized and palpated in the area of the normotrophic, uncomplicated cicatrix from the previous surgery.

**Figure 3:**
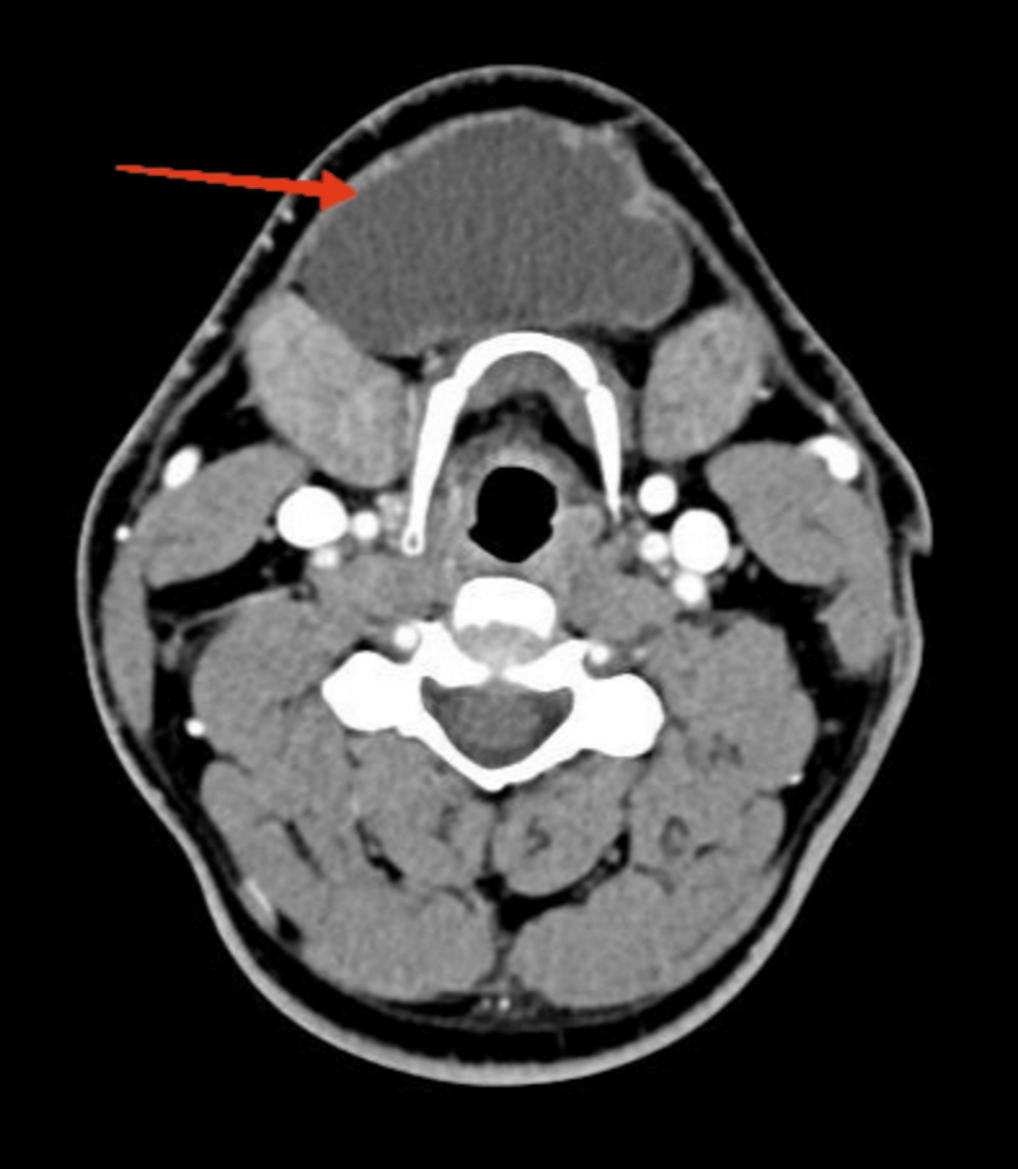
Sagittal section of the CT with intravenous administration of contrast material, showing a fluid-equivalent mass well demarcated from the surrounding parenchyma, without peripheral enhancement. It is located medially in the submental space, in close proximity to the right sublingual gland, without communication with the left sublingual gland CT, computed tomography

**Figure 4:**
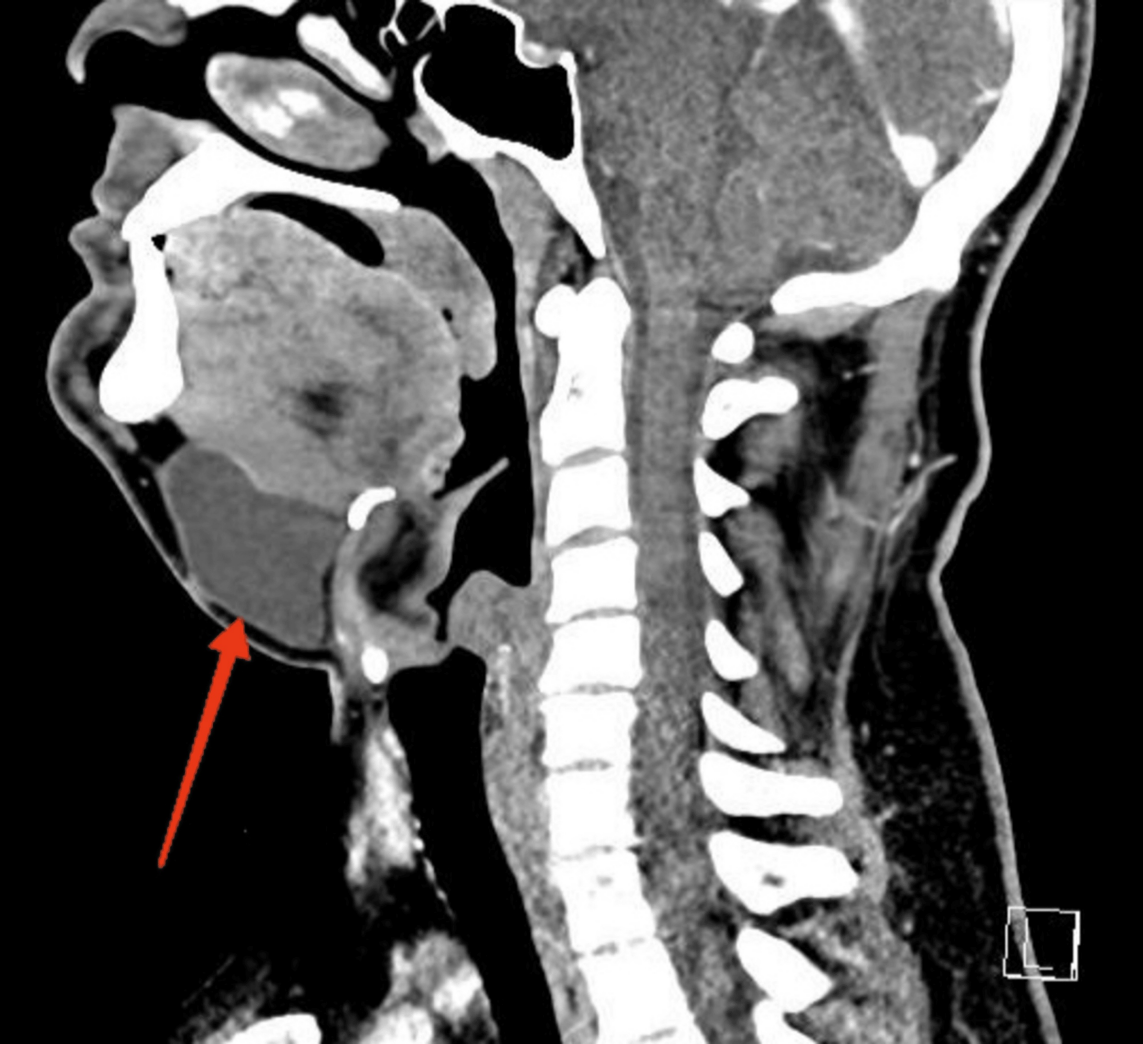
Axial CT image with intravenous contrast material showing a fluid-equivalent mass well demarcated from the surrounding parenchyma, with no peripheral enhancement. It is located medially in the submental space and cranially reaches the caudal surface of the mylohyoid muscle CT, computed tomography

Contrast-enhanced CT of the head and neck was performed, which showed a fluid-equivalent lesion with a native and post-contrast density of up to 18-20HU, located medially in the submental space, in close proximity to the right sublingual gland. The border between the two was represented solely by the mylohyoid muscle, and the finding did not communicate with the left sublingual gland. The described finding had axial dimensions of up to 62/28 mm and craniocaudal size of up to 50 mm. Cervical lymph nodes were normal (Figures 3, 4).

The patient was scheduled for repeat surgery to remove the current swelling. A similar surgical approach was utilized. A cyst-like lesion located in the same area and with similar characteristics - a thin and easily rupturable wall and a size of about 5 cm in diameter - was removed in an identical manner under general anesthesia. It was sent for a histological examination. The histopathological findings were identical to those from the previous excision, and the diagnosis again was a plunging ranula.

The patient was discharged on the third postoperative day without surgical complications. Given the recurrent nature of the process, he was offered surgical extirpation of the right sublingual salivary gland, which, according to the available CT data (Figures 3, 4), was assumed to be the cause of the recurrent disease. Despite the proposed treatment, the patient is still considering his decision to date. During this time, he was under regular observation with an uneventful postoperative period, normal wound healing, no complaints, and no physical evidence of recurrence.

## Discussion

Ranulas are cystic lesions that affect the sublingual salivary gland and occur most often as a result of obstruction of normal salivary flow as a result of trauma, glandular ectopia, or of unknown etiology [7,8]. Saliva extravasation in plunging ranulas can reach up to the base of the skull and down through the soft tissues of the neck to the supraclavicular region and even enter the mediastinum [7]. Most often it affects the submental space, which is usually caused by herniation of the mylohyoid muscle or ectopically located parts of the sublingual gland [8]. From the performed ultrasound and CT in the patient presented by us, there was no evidence of ectopism of the major salivary glands, and we assumed that the extravasation of saliva, in this case, was caudal to the mylohyoid muscle through the muscle fibers themselves.

Plunging ranulas are usually asymptomatic lesions, presenting with a progressively enlarging swelling, as in the current patient [7,8]. The masses, including in this patient, have a soft texture and are significantly mobile and painless [9,10]. They can appear at any age but are most common from the first to the sixth decades of life [10]. As exceptions, Mahadevan and Vasan described cases of children younger than ten years of age with diagnosed plunging ranula, and Matt and Crockett described a case in a two-month-old child [11,12]. They more often affect the female gender [11,12]. According to Langlois and Kolhe, the ratio between the two sexes is approximately 3:1 in favor of women [7,8,10]. The etiology of the disease remains incompletely understood, with possible causes being congenital anomalies, trauma, and other diseases of the salivary gland [8]. Some authors, such as Zdilla et al., describe a possible genetic component as an etiological cause [13].

In the patient we present, the possible etiology still remains unclear - he lacks both physical and imaging evidence of ectopism of major salivary glands, as well as anamnestic evidence of genetic disorders and head and neck trauma.

The diagnosis of this disease requires correct clinical evaluation as well as considerable involvement of paraclinical methods. Paraclinical studies suitable for this purpose are MRI, CT, ultrasound, FNA, and histological analysis of the excised material [14].

The differential diagnosis includes a number of benign and malignant diseases - lesions of the sublingual and submandibular salivary glands, epidermoid and dermoid cysts, branchial and thyroglossal duct cysts, and laryngocele. The similar clinical features and localization of thyroglossal duct cysts and plunging ranulas create significant difficulties during the diagnostic process [15]. This initially occurred in the present patient, in whom, prior to the first operative intervention (in September 2023), both the physical and sonographic characteristics of the lesion suggested a thyroglossal duct cyst, and this was our initial clinical diagnosis.

There are some limitations of ultrasonography in establishing a correct diagnosis, such as the insufficient penetrating power of various transducers, the many artifacts of surrounding tissues, and the experience of the observer, which can cause errors in differentiating a plunging ranula from a median cyst and other types of fluid-equivalent lesions. For these reasons, CT and MRI are advantageous as diagnosis methods, allowing better visualization of the pathological process, better spatial orientation (which in turn would allow the surgeon to precisely plan the operative volume), more accurate determination of the depth of invasion, and also application of contrast material in order to characterize the pathological finding itself.

The histopathological examination is the gold standard in terms of differentiating between thyroglossal duct cysts and plunging ranula. Histologically, a thyroglossal duct cyst is a true cyst with epithelial lining of either respiratory or squamous epithelium or most commonly combination of both. Presence of ectopic thyroid tissue is seen in 31-62% of cases [16]. The absence of any of those findings in the present case precludes the diagnosis of median cyst, and plunging ranula was accepted.

Surgical excision of the lesion along with the affected salivary gland is the gold standard of treatment for these types of retention cysts and includes incision and drainage of the cystic sac, marsupialization, and total or partial sialoadenectomy [9,17]. Methods such as marsupialization and incision and drainage are not preferred in the treatment of plunging ranulas because recurrence is then observed in 61-89% of cases [9,17]. According to Roh, the total extirpation of the sublingual gland together with the cyst itself leads to no recurrence in about 98% of cases [18]. He claims that only 4-38% of patients undergoing marsupialization or incision and drainage of the lesion remain recurrence-free [18]. According to the same author, partial sialadenectomy, including removal of the cyst, leads to recurrence in about 25% of patients [18].

The current patient was offered a third operative treatment - a total extirpation of the right sublingual gland, which, given the CT data, was supposed to be the causative factor of his condition, but he refused it. Expectedly, recurrence occurred approximately seven months later and resulted in a second operation in March 2024. Since then, for five months now, the patient has been without complaints and without physical evidence of recurrence from regular postoperative examinations and continued to refuse further surgical treatment.

## Conclusions

Although rare, plunging ranulas can be the cause of a series of operations aimed at their surgical removal, especially if they are not initially diagnosed correctly. Most often, a differential diagnosis is made with thyroglossal cysts, which have similar clinical and imaging characteristics and can mislead even the most experienced clinician, as happened when making the preliminary diagnosis of the patient we presented. However, the pathological analysis of the excised material is usually definitive and points to the correct final diagnosis. It is of great importance to conduct the correct imaging investigations leading to the correct diagnosis of plunging ranula, contrast-enhanced CT or MRI of the head and neck.

## References

[1] Jing F, Wu F, Wen Y, Gao Q (2023). Plunging ranula presenting as a giant anterior cervical cystic mass: a case report and literature review. Case Rep Oncol.

[2] Kolomvos N, Kalfarentzos E, Papadogeorgakis N (2019). Surgical treatment of plunging ranula: report of three cases and review of literature. Oral Maxillofac Surg Cases.

[3] Koch M, Mantsopoulos K, Leibl V, Müller S, Iro H, Sievert M (2023). Ultrasound in the diagnosis and differential diagnosis of enoral and plunging ranula: a detailed and comparative analysis. J Ultrasound.

[4] Suresh BV, Vora SK (2012). Huge plunging ranula. J Maxillofac Oral Surg.

[5] Zhao YF, Jia Y, Chen XM, Zhang WF (2004). Clinical review of 580 ranulas. Oral Surg Oral Med Oral Pathol Oral Radiol Endod.

[6] Olojede AC, Ogundana OM, Emeka CI (2018). Plunging ranula: surgical management of case series and the literature review. Clin Case Rep.

[7] Kalra V, Mirza K, Malhotra A (2011). Plunging ranula. J Radiol Case Rep.

[8] de Visscher JG, van der Wal KG, de Vogel PL (1989). The plunging ranula: pathogenesis, diagnosis and management. J Craniomaxillofac Surg.

[9] Morton RP (2018). Surgical management of ranula revisited. World J Surg.

[10] Lomas J, Chandran D, Whitfield BC (2018). Surgical management of plunging ranulas: a 10-year case series in South East Queensland. ANZ J Surg.

[11] Mahadevan M, Vasan N (2006). Management of pediatric plunging ranula. Int J Pediatr Otorhinolaryngol.

[12] Matt BH, Crockett DM (1988). Plunging ranula in an infant. Otolaryngol Head Neck Surg.

[13] Malpas P (1926). Anomalies of the mylohyoid muscle. J Anat.

[14] Yun J, Gidumal S, Saturno MP (2024). Diagnostic difficulties of plunging ranula: a review of 18 cases. Laryngoscope.

[15] Shelley MJ, Yeung KH, Bowley NB, Sneddon KJ (2002). A rare case of an extensive plunging ranula: discussion of imaging, diagnosis, and management. Oral Surg Oral Med Oral Pathol Oral Radiol Endod.

[16] Thompson LD, Herrera HB, Lau SK (2016). A clinicopathologic series of 685 thyroglossal duct remnant cysts. Head Neck Pathol.

[17] Morita Y, Sato K, Kawana M, Takahasi S, Ikarashi F (2003). Treatment of ranula - excision of the sublingual gland versus marsupialization. Auris Nasus Larynx.

[18] Roh JL (2022). Transoral complete vs partial excision of the sublingual gland for plunging ranula. Otolaryngol Head Neck Surg.

